# Utility of a partially covered metal stent for salvage sealing therapy for bleeding caused by duodenal invasion of pancreatobiliary cancers: Case series

**DOI:** 10.1002/deo2.253

**Published:** 2023-06-09

**Authors:** Yasunobu Yamashita, Hirofumi Yamazaki, Yuki Kawaji, Takashi Tamura, Keiichi Hatamaru, Masahiro Itonaga, Reiko Ashida, Masayuki Kitano

**Affiliations:** ^1^ Second Department of Internal Medicine Wakayama Medical University Wakayama Japan

**Keywords:** advanced pancreatobiliary cancer, case series, covered self‐expandable metal stent, gastrointestinal bleeding, novel technique

## Abstract

Pancreatobiliary cancer‐related gastrointestinal bleeding caused by duodenal invasion can be a life‐threatening condition that is hard to control. It is unclear whether a covered self‐expandable metal stent (CSEMS) is useful for hemostasis of bleeding related to advanced pancreatobiliary cancer. The aim of this study was to evaluate the utility of a CSEMS for hemostasis of bleeding caused by duodenal invasion of pancreatobiliary cancer. Between January 2020 and January 2022, seven patients in whom a duodenal CSEMS was inserted to control pancreatobiliary cancer‐related bleeding were enrolled. The technical and clinical success rates with respect to hemostasis, procedure time, and adverse events were assessed. All patients were inoperable cases (six with pancreatic cancer [five, stage IV; one, stage III]; and one with gallbladder cancer [stage IV]) in whom CSEMs were inserted to treat refractory bleeding caused by cancer invasion. Hemostasis was achieved in all cases (100% [7/7]). The mean procedure time was 17 ± 7.9 min. There were no adverse events, including migration and rebleeding. No rebleeding occurred up until the time of death in any of the cases (mean follow‐up period, 73 ± 27 days). Deployment of duodenal CSEMS is a useful salvage therapy for bleeding caused by advanced pancreatobiliary cancer invasion.

## INTRODUCTION

Pancreatobiliary cancer has a poor prognosis.^1^, ^2^ Moreover, most cases of pancreatic cancer are discovered at an unresectable stage; indeed, the 5‐year survival rate is only 11%.^2^ Overall, 35.7% of all pancreatic cancer patients suffer from duodenal infiltration^3^, and the primary manifestation in 2.6% of patients is gastrointestinal bleeding.^4^ Massive hemorrhage might lead to an oncological emergency. Gastrointestinal bleeding is treated by endoscopic hemostasis, transcatheter arterial embolization, or surgery. Although pancreatic cancer is a rare cause of gastrointestinal bleeding, endoscopic approaches are not really suitable for stopping bleeding caused by pancreatic cancer invasion due to the associated duodenal narrowing, and to the fact that the cancer tissue itself is fragile. Moreover, once bleeding has occurred, it often recurs, resulting in serious blood loss.^5^, ^6^, ^7^ Failure of hemostasis results in a poor prognosis because it is difficult to continue cancer chemotherapy due to gastrointestinal bleeding. Nowadays, a covered self‐expandable metal stent (CSEMS) is used to stop refractory bleeding, particularly refractory variceal bleeding. The aim of this case series was to assess the utility of CSEMS for preventing bleeding caused by duodenal invasion by advanced pancreatobiliary cancers.

## CASE REPORT

Between January 2020 and January 2022, seven patients who received duodenal SEMS to treat pancreatobiliary cancer‐related bleeding were enrolled. A therapeutic flowchart is shown in Figure S1.

Endoscopy using a GIF‐2T240 (Olympus, Tokyo, Japan) was performed by endoscopy specialists. First, the bleeding site was checked. Second, a guidewire was inserted over the stenosis and bleeding area. Partially covered braided stents (Niti‐S & ComVi Pyloric/Duodenal Stent: TaeWoong Medical, Gimpo, Korea; Century Medical Inc., Tokyo, Japan) were used (Figure S2). The length of the covered part of the SEMS overlaid the site of bleeding and cancer invasion.

All patients had advanced cancer. Two patients (29%) were taking antiplatelet or antithrombotic drugs, and three had received a transfusion due to bleeding [two, moderate (up to 4 units); one, severe (>5 units)] (Table 1). However, none of the patients had gone into shock due to blood loss. The mean procedure time was 17 ± 7.9 min, and there were no adverse events, including stent migration and rebleeding. After the procedure, the patients were able to eat meals about 2 ± 1 days later. Follow‐up endoscopic findings, clinical symptoms, and laboratory data regarding anemia suggested that hemostasis was successful in all cases (100%). No rebleeding was observed in any of the cases (mean follow‐up period, 73 ± 27 days). Endoscopic ultrasound (EUS)‐guided biliary drainage (EUS‐BD) was performed in one case before CSEMS insertion and in two cases after CSEMS insertion. Five of the seven patients died due to cancer progression (Table 2). No rebleeding occurred up until the time of death. The median overall survival after the initial episode of gastrointestinal bleeding was 90 days (95% CI 42–; Figure S3).

**TABLE 1:** Background and clinical characteristics of the patients receiving a covered self‐expandable metal stent to prevent bleeding caused by duodenal invasion by pancreatobiliary cancer.

No.	Age	Sex	Diagnosis	Stage	Antiplatelet/antithrombotic drugs	Shock vital	Change in Hb level, g/dL	Blood transfusion (units)
1	71	M	Pancreatic cancer	IV	−	−	−1.1	−
2	54	F	Pancreatic cancer	IV	−	−	−1.2	−
3	50	M	Pancreatic cancer	III	−	−	−1.4	−
4	70	F	Pancreatic cancer	IV	−	−	−3.7	+ (8 U)
5	51	F	Gallbladder cancer	IV	+	−	−2.1	+ (4 U)
6	93	F	Pancreatic cancer	IV	−	−	−2.4	+ (4 U)
7	68	M	Pancreatic cancer	IV	+	−	−1	−

**TABLE 2:** Type of covered self‐expandable metal stent, and outcomes of covered self‐expandable metal sten deployment to prevent bleeding caused by duodenal invasion by pancreatobiliary cancer.

No.	Type of CSEMS	Prior uncovered SEMS	Length of CSEMS (mm)	Procedure time (min)	Technical success	Successful hemostasis	Adverse events	Rebleeding	Meal starts period after stent insertion (days)	Follow‐up period (days)	Death
1	Niti‐S and ComVi stent	+	120	16	+	+	−	−	3	95	+
2	Niti‐S and ComVi stent	+	120	9	+	+	−	−	1	90	+
3	Niti‐S and ComVi stent	+	120	30	+	+	−	−	1	121	+
4	Niti‐S and ComVi	+	120	13	+	+	−	−	2	42	+
5	Niti‐S and ComVi stent	+	100	17	+	+	−	−	1	60	−
6	Niti‐S and ComVi stent	+	120	12	+	+	−	−	3	44	−
7	Niti‐S and ComVi stent	+	120	27	+	+	−	−	3	60	+

### Case 1 (No.1)

**FIGURE 1:**
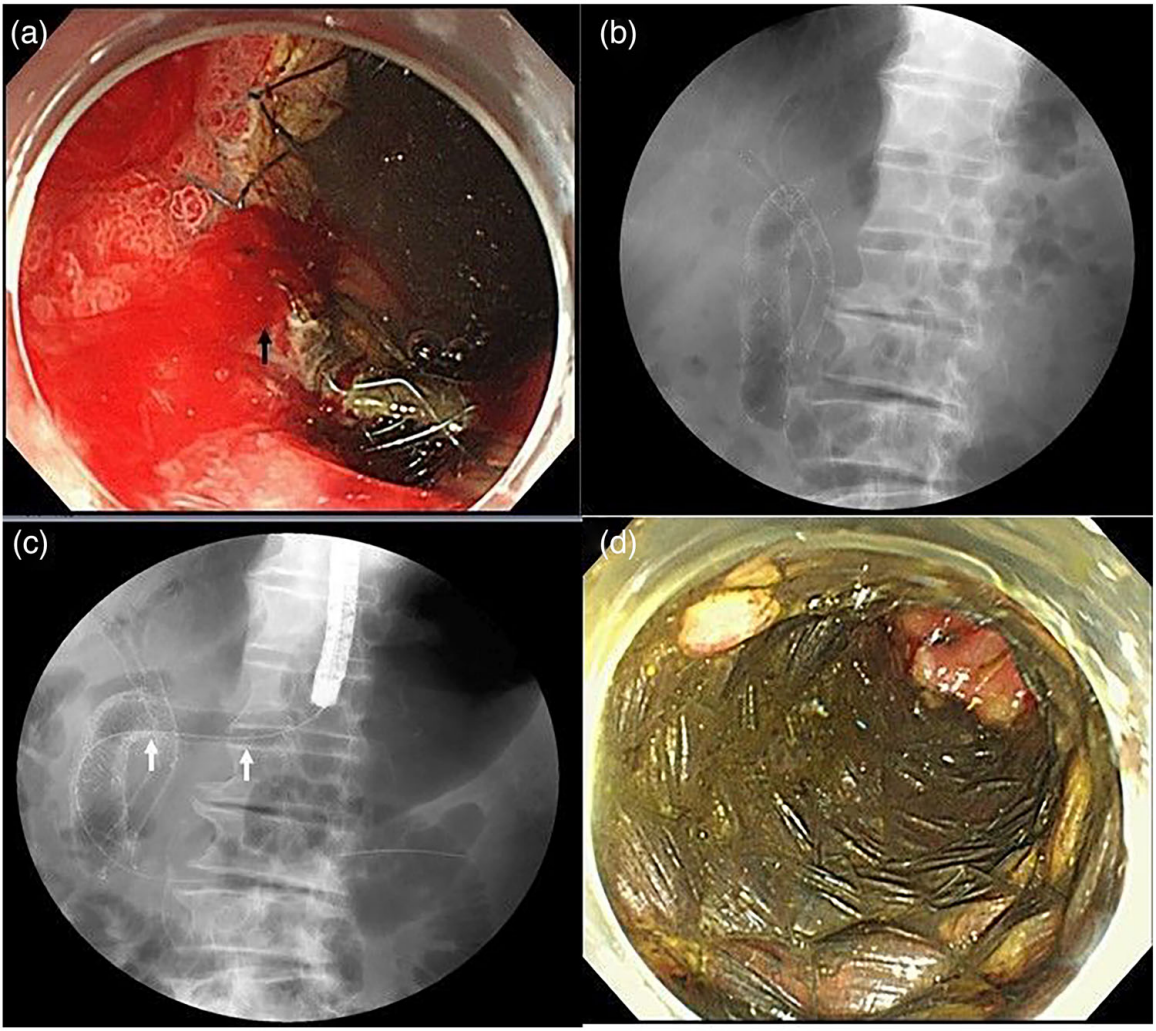
Images from one patient (No. 1) oozing from fragile cancer tissue. (a) Oozing from the edge of the prior uncovered self‐expandable metal stent was observed at the bulbs of the duodenum. (b) Patient had undergone placement of an uncovered self‐expandable metal stent for duodenal stenosis. (c) A duodenal stent (20 mm wide and 120 mm long) was inserted such that the membrane‐covered portion of the stent covered the oozing area (arrow). (d) No rebleeding was observed upon re‐evaluation performed the next day after the procedure.

A 71‐year‐old man visited an emergency unit due to a tarry stool. Emergency endoscopy was performed, and oozing from the edge of prior uncovered SEMS (USEMS) was observed at the bulbs of the duodenum. It was necessary to place the stent from the stomach to the duodenum because tumor infiltration was detected near the pyloric ring in the bulbs of the duodenum. No rebleeding was observed upon re‐evaluation (Figure 1).

### Case 2 (No.5)

**FIGURE 2:**
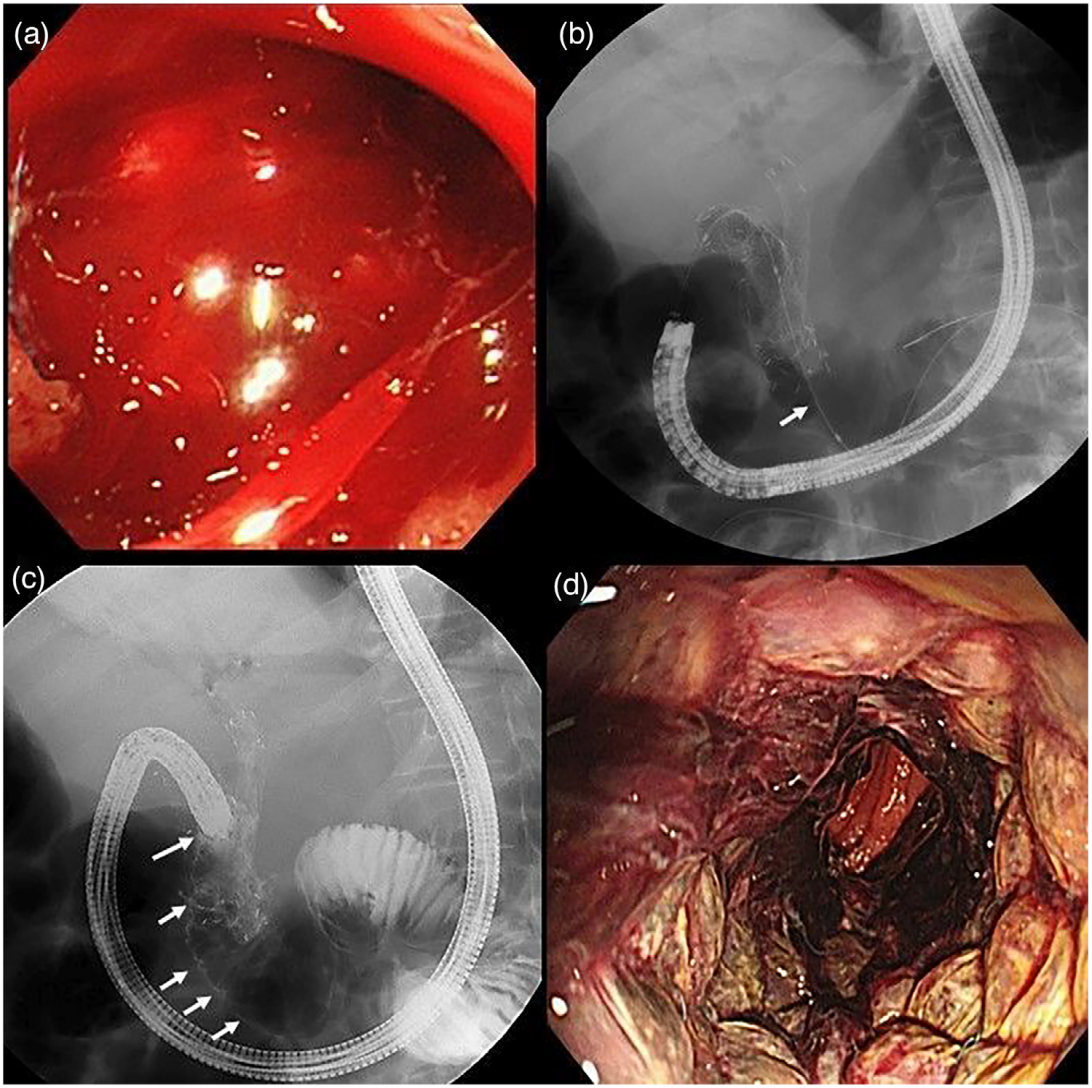
Images from one patient (No. 5) with bleeding caused by cancer invasion. (a) Bleeding and stenosis were observed from the bulbs to the second portion. (b) A guidewire (arrow) was inserted over the area of stenosis and bleeding. (c) A duodenal stent (20 mm wide and 120 mm long) was inserted such that the membrane‐covered portion of the stent covered the bleeding and stenosis area (arrow). (d) No rebleeding was observed upon re‐evaluation performed 3 days after the procedure.

A 51‐year‐old woman visited an emergency unit due to melena and the progression of anemia. Emergency endoscopy was performed, and although bleeding caused by cancer invasion at the site of the previous stent insertion was detected, the exact bleeding site was difficult to isolate. The bleeding continued on the next day; therefore, we decided to deploy a CSEMS inside the USEMS. A guidewire was inserted over the site of cancer invasion and bleeding, followed by the deployment of a CSEMS. We then checked and confirmed hemostasis. We confirmed hemostasis and no progression of anemia (Figure 2).

## DISCUSSION

The seven cases presented herein demonstrate that the insertion of a CSEMS is a viable therapeutic option for bleeding caused by pancreatobiliary cancer invasion. Duodenal bleeding occurs in 1.6% of pancreatic cancer cases; of these, 73.6% have metastatic or locally advanced disease.^8^ Although duodenal bleeding in patients with pancreatic cancer is not common, it is associated with a very poor short‐term prognosis. Indeed, the median overall survival from the episode of gastrointestinal bleeding is 2.7 months.^9^ Five of the seven patients reported herein died without rebleeding due to the progression of cancer after the episode of gastrointestinal bleeding; the median overall survival time after bleeding was 90 days.

In general, three hemostatic methods are used to prevent bleeding: endoscopic hemostasis, transcatheter arterial embolization, and/or surgery. Although transcatheter arterial embolization is useful to stop arterial bleeding, it is not indicated for venous bleeding caused by cancer invasion. Surgical hemostasis, including pancreaticoduodenectomy, is extremely invasive for patients with highly advanced cancers. For these patients, endoscopic hemostasis plays an important role in preventing gastrointestinal bleeding. Moreover, duodenal invasion causes not only bleeding but also duodenal obstruction, for which treatment is necessary. In fact, all seven cases had undergone placement of a USEMS for duodenal stenosis; bleeding was caused by infiltration of cancer cells through the mesh of the USEMS. Therefore, endoscopic hemostasis is a reasonable method because stenosis can be treated as well. However, the procedure might be very difficult when cancer infiltration narrows the duodenal lumen, as this restricts the endoscopic view and the ability to maneuver the scope. Moreover, the fragile cancer tissue might impede endoscopic hemostasis achieved by clipping or focal injection of hypertonic saline/epinephrine. Therefore, other endoscopic hemostasis procedures are needed under such circumstances.

CSEMS is used for endoscopic hemostasis under difficult conditions and the procedure is a safe and effective alternative approach as a rescue therapy for refractory variceal bleeding. A meta‐analysis of 12 studies revealed that the pooled technical success rate and clinical success rate for treating esophageal variceal bleeding with a CSEMS was 97% (95% CI 0.91–100) and 96% (95% CI 0.9–1.0), respectively; the overall rate of adverse events was 36%. The most common adverse event was stent migration (23%). The rate of rebleeding due to stent migration was 7%.^10^ Therefore, a CSEMS is a useful hemostatic method. When patients with duodenal stenosis did not display extravasation of contrast media on a computed tomography scan and endoscopic hemostasis such as injectable, thermal, or mechanical therapy was difficult, a duodenal CSEMS was deployed for hemostasis. The rate of stent migration is lower in cases of bleeding caused by pancreatobiliary cancer invasion than in cases of gastrointestinal bleeding due to other causes because duodenal tumor invasion results in duodenal stenosis. In fact, all of our cases had duodenal stenosis. We used a partial CSEMS because the rate of stent migration is lower than that for a full CSEMS. The partial CSEMS that we used also had a flare stricture to prevent migration. In our study, the technical and clinical success rates were 100% and 100%, respectively. The rate of adverse events, including stent migration and rebleeding, was 0%. The mean procedure time was 17 ± 7.9 min. The advantages of this approach are as follows: First, it is possible to stop bleeding without identifying the exact site of the bleeding. This is particularly useful in highly difficult situations, such as lumen narrowing and the presence of fragile cancer tissue, because the stent covers all of the duodenal mucosa that is bleeding. Second, this method allows treatment of duodenal stenosis. Third, the medical costs for this approach (92100 yen) are lower than those for transcatheter arterial embolization (231100 yen) and surgery (113600 yen).

In conclusion, the insertion of a partial CSEMS is a useful salvage therapy for bleeding caused by duodenal invasion by advanced pancreatobiliary cancer. The method may have advantages such as feasibility, reduced medical costs, and hemostatic efficacy.

## CONFLICT OF INTEREST STATEMENT

Masayuki Kitano has received honoraria from Olympus Corporation for giving lectures at conferences. The other authors declare no conflict of interest.

## Supporting information


**Supplementary Figure 1**. Therapeutic flow‐chart for pancreatobiliary cancer‐related bleeding. CSEMS, covered self‐expandable metal stent.

**Supplementary Figure 2**. A partially covered self‐expandable metal stent. The braided expanded stent is 20 mm in diameter. The main part of the stent is covered with a polytetrafluoroethylene (PTFE) membrane, with a 15 mm section at each end being uncovered. The proximal end is equipped with flare 25 mm wide.

**Supplementary Figure 3**. Overall survival after the episode of gastrointestinal bleeding.
