# Supplementary figures and images for: Utility of a partially covered metal stent for salvage sealing therapy for bleeding caused by duodenal invasion of pancreatobiliary cancers: Case series
Source: DEN Open. 2023 Jun 9;4(1):e253. doi: 10.1002/deo2.253 (PMC10256867; doi:10.1002/deo2.253)

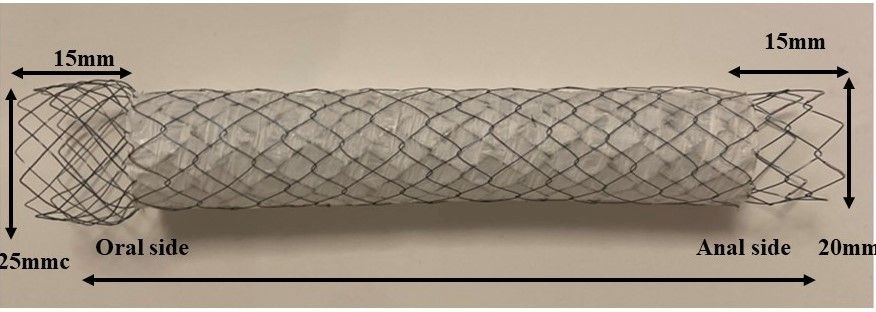

Supplement: Supplementary file 1 — Supplementary Figure 1. Therapeutic flow‐chart for pancreatobiliary cancer‐related bleeding. CSEMS, covered self‐expandable metal stent. [file DEO2-4-e253-s002.jpg]

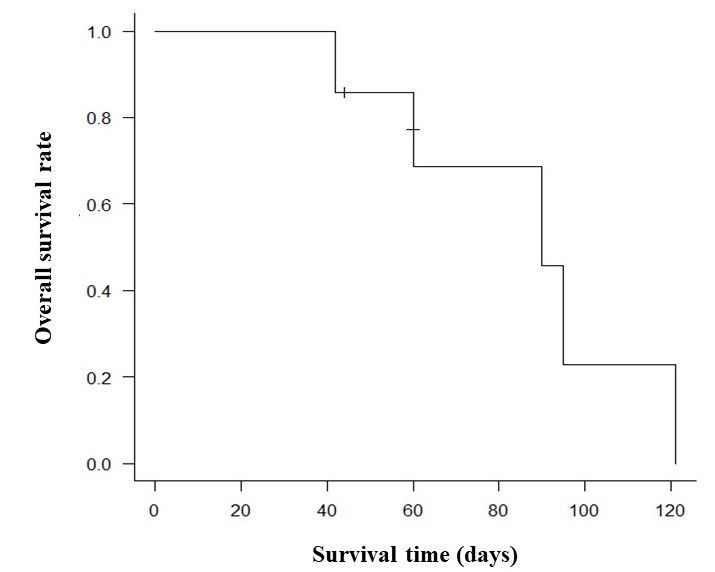

Supplement: Supplementary file 2 — Supplementary Figure 2. A partially covered self‐expandable metal stent. The braided expanded stent is 20 mm in diameter. The main part of the stent is covered with a polytetrafluoroethylene (PTFE) membrane, with a 15 mm section at each end being uncovered. The proximal end is equipped with flare 25 mm wide. [file DEO2-4-e253-s001.jpg]

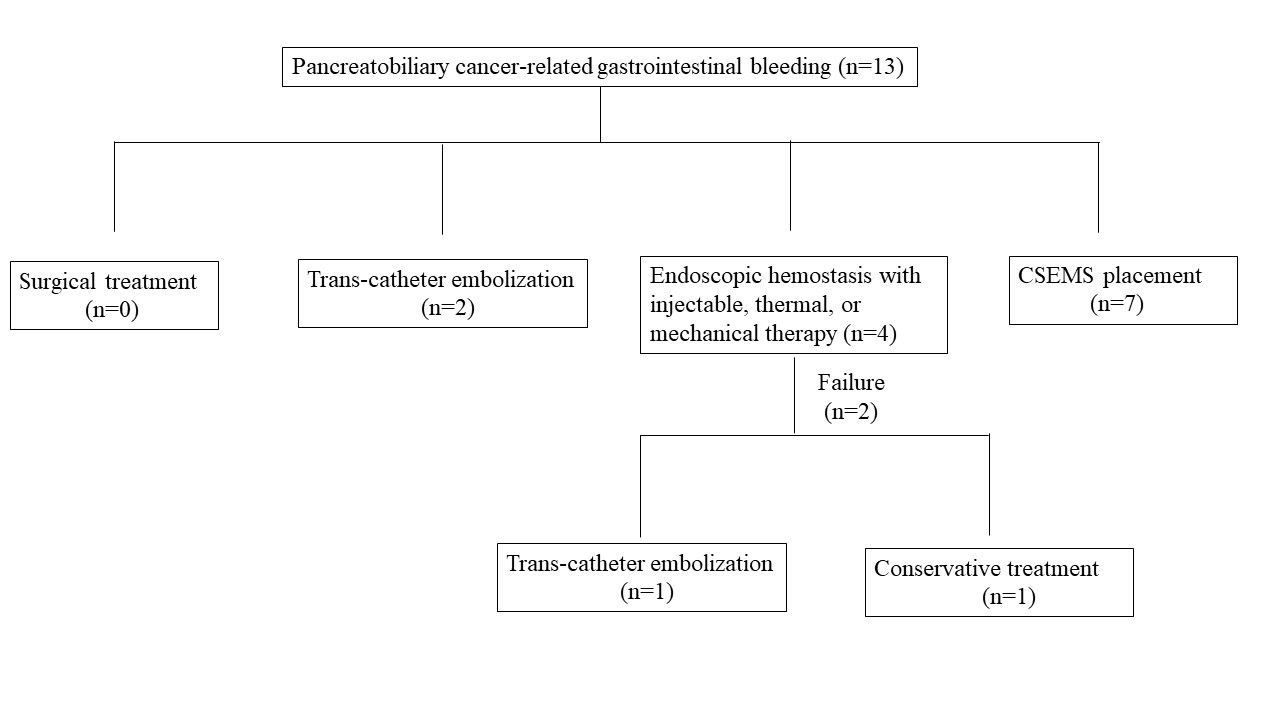

Supplement: Supplementary file 3 — Supplementary Figure 3. Overall survival after the episode of gastrointestinal bleeding. [file DEO2-4-e253-s003.jpg]
